# Targeting NR4A1 (Nur77) in Macrophage Polarization: Molecular Mechanisms and Translational Prospects for Cardiac Repair After Myocardial Infarction

**DOI:** 10.3390/ijms27177774

**Published:** 2026-08-30

**Authors:** Jiaqian Lin, Jianmin Wang, Lei Sun, Rui Zhang

**Affiliations:** 1 Department of Pathology and Forensic Medicine, College of Basic Medical Sciences, Dalian Medical University, Dalian 116044, China; linjiaqian8383@163.com; 2 Department of Science and Technology, Dalian Medical University, Dalian 116044, China; wjm_0902@163.com

**Keywords:** myocardial infarction, macrophage polarization, NR4A1 (Nur77), inflammation resolution, cardiac repair

## Abstract

Sterile inflammation in the course of myocardial infarction can be a factor contributing to cardiac remodeling and heart failure. To break this pathological vicious circle, the polarization of macrophages to a reparative M2 phenotype, to start the tissue repair process and alleviate the inflammation, is the key. Recent studies have shown that the nuclear receptor subfamily 4 group A (NR4A) orphan nuclear receptor family, particularly nuclear receptor subfamily 4 group A member 1 (NR4A1/NUR77), plays a role in macrophage reprogramming in relation to the modulation of macrophage polarization. Herein, we discuss in detail the specific pathways regulated by NR4A receptors during cellular reprogramming, including the nuclear factor kappa B (NF-κB) pathway and the oxidative phosphorylation pathway. We list below the beneficial effects of NR4A-treated macrophages in the hypoxic cardiac tissue, including their essential contribution to the formation of a neovascular network, the maintenance of the extracellular matrix by fibroblasts, and the direct rescue of remaining cardiomyocytes. In addition, given the translational potential of targeted therapy with immunomodulatory molecules against NR4A, we also review the design of macrophage-specific nanodelivery systems and the creation of novel small-molecule activators for ischemic cardiovascular diseases to identify therapeutic targets for cardiac repair based on NR4A.

## 1. Introduction

Despite considerable progress in interventional cardiology, myocardial infarction (MI) and its progression to heart failure remain among the most lethal cardiovascular events globally [1]. Beyond the immediate loss of cardiomyocytes from ischemic injury, the injured myocardium triggers a vigorous wave of sterile inflammation fueled by endogenous danger signals from dying cells [2]. Timely reperfusion improves survival, yet the ischemia–reperfusion (I/R) process itself aggravates injury and produces an inflammatory environment that macrophage polarization largely shapes [3]. Macrophages are the most abundant and functionally diverse of the cardiac resident immune cell populations, and they coordinate both steady-state immune surveillance and the response to injury [4]. Their defining feature is an ability to switch between distinct activation states as local tissue signals change [5]. Danger signals such as damage-associated molecular patterns (DAMPs) or classical Th1-derived stimuli such as lipopolysaccharide (LPS) and interferon-γ (IFN-γ) drive macrophages toward a pro-inflammatory M1 phenotype marked by heightened phagocytic activity and strong secretion of signature cytokines [6,7]. Th2-related cytokines, interleukin-4 (IL-4) in particular, polarize them instead to M2, which suppresses inflammation and promotes tissue repair [8]. After myocardial infarction, M1 macrophages carry the initial inflammatory response and the phagocytosis of cell debris, which sets the stage for the later phases of healing. Later, there should be a timely switch to M2, reparative macrophages, to prevent excessive fibrosis and the onset of maladaptive ventricular remodeling [9,10,11]. However, if inflammation is prolonged, it hinders the ability of the tissue to heal and encourages it to remodel in the wrong way [12]. Recently, evidence has emerged that the NR4A orphan nuclear receptor family (NR4A1, NR4A2, and NR4A3) plays an important role in regulating macrophage plasticity. Preclinical studies have implicated NR4A receptors in macrophage phenotypic reprogramming through suppression of inflammatory gene expression and modulation of cellular metabolism toward oxidative phosphorylation [13,14]. As NR4A1 (Nur77) is the most well-studied NR4A family member in cardiac macrophage biology, this review mainly focuses on a comprehensive understanding of the molecular mechanisms by which NR4A1 (Nur77) regulates macrophage phenotype switching and cardiac repair after myocardial infarction (MI). Although evidence is scarce for nuclear receptor subfamily 4 group A member 2 (NR4A2/Nurr1) and nuclear receptor subfamily 4 group A member 3 (NR4A3/NOR-1), they are also discussed in detail where appropriate in this review.

## 2. Literature Search Strategy

A structured literature search was conducted using PubMed, with the final search performed on 16 August 2026. Search terms included combinations of “NR4A,” “NR4A1,” “Nur77,” “NR4A2,” “Nurr1,” “NR4A3,” “NOR-1,” “macrophage,” “monocyte,” “myocardial infarction,” “cardiac repair,” “cardiac remodeling,” “atherosclerosis,” “cardiac fibrosis,” “efferocytosis,” “NF-κB,” “CoREST,” “oxidative phosphorylation,” and related terms. Additional targeted searches were performed for mechanistically relevant cellular contexts and therapeutic approaches. No publication-date restriction was applied, and preprints were excluded. Priority was given to peer-reviewed original studies directly examining NR4A signaling in macrophages or monocytes, experimental cardiovascular disease, myocardial injury and repair, or mechanistically relevant cellular systems. Studies from non-cardiac contexts were included when they provided mechanistic evidence relevant to NR4A-dependent immune regulation or macrophage biology. Relevant review articles and key primary studies were also screened to identify additional original studies through reference-list screening. Because this article is a narrative review rather than a systematic review, references were selected according to their relevance to the scope of the review, and the literature was synthesized qualitatively without formal meta-analysis.

## 3. NR4A Family Members

The NR4A subfamily consists of the three orphan receptors NR4A1 (Nur77/TR3), NR4A2 (Nurr1), and NR4A3 (NOR-1) and belongs to Group 4 of the nuclear receptor superfamily [15]. Structurally, they are unlike the traditional nuclear receptors, where the ligand-binding domain has a pocket for binding to a small-molecule ligand—hence the term “orphan” receptors [16]. Consequently, their transcriptional activity is not ligand-dependent but rather is induced by rapid transcriptional induction and is controlled by post-translational modifications [17]. The NR4A family has recently garnered significant attention due to its role in cardiovascular diseases. Although largely structurally similar, each member has unique but complementary functions in maintaining cardiovascular and immune homeostasis [18]. In experimental atherosclerosis models, NR4A1 (Nur77) has been shown to restrain pro-inflammatory macrophage activation and limit lesion progression [19]. However, its effects are context-dependent and vary with the tissue environment and pathological stimulus. Evidence from experimental studies suggests that NR4A2 (Nurr1) can modulate macrophage lipid metabolism and inflammatory responses in the context of atherosclerosis [18]. Experimental studies of vascular injury have implicated NR4A3 (NOR-1) in vascular smooth muscle-cell proliferation and immune-cell localization during vascular remodeling [20].

NR4A receptors are only expressed at low basal levels in several cardiovascular cell types, such as cardiomyocytes, resident macrophages, vascular smooth muscle cells, and fibroblasts, under normal physiological conditions. These NR4A receptors, in these cells, regulate metabolic balance and quiescence in cardiovascular tissues [21]. This balance is, however, quickly disrupted by MI, and NR4A expression is significantly increased in a cell-type-specific manner [22]. The NR4A family members are members of the immediate early gene family and are rapidly activated following ischemic insults, oxygen deprivation, and sterile inflammatory triggers [23]. Molecularly, the extreme hypoxia in the ischemic region leads to diminished degradation of hypoxia-inducible factor 1 alpha (HIF-1α), which is normally dependent on oxygen; thus, HIF-1α stability is increased, and it aggregates not only in cardiomyocytes but also in infiltrating immune cells. The sum of HIF-1α translocates to the nuclei, where it binds hypoxia response elements (HREs) present in promoter regions of NR4A genes and consequently activates their transcription [15]. At the same time, acute ischemic stress activates the sympathetic nervous system, resulting in an increase in the release of catecholamines that activate NR4A expression through the β-adrenergic/cyclic adenosine monophosphate (cAMP)/protein kinase A (PKA) signaling pathway in macrophages and in cardiomyocytes, where it drives metabolic adaptation [24]. Moreover, DAMPs released from necrotic cardiomyocytes engage Toll-like receptors (TLRs) on infiltrating monocytes and macrophages, which rapidly activate downstream signaling cascades such as p38 mitogen-activated protein kinase (MAPK) and NF-κB, causing NR4A mRNA levels to peak dramatically within 1 to 2 h after injury onset, with the most prominent upregulation occurring in macrophages [9,25].

Through multi-pathway, synergistic induction, NR4A receptors are positioned as highly sensitive stress sensors within the post-infarct microenvironment, rapidly converting acute damage signals to modulate the transcriptional reprogramming of infiltrating and resident macrophages [26]. NR4A receptors do not merely register stress signals. They act as “molecular switches,” converting cues from the microenvironment into substantial reprogramming of intracellular signaling [16]. Their role reaches past the classical functions in apoptosis and proliferation: the family is increasingly viewed as a set of master regulators of macrophage metabolic remodeling and inflammatory homeostasis [27]. In experimental macrophage models, NR4A1 (Nur77) has been shown to reprogram mitochondrial metabolism, including succinate/succinate dehydrogenase (SDH)-associated pathways, and in doing so, it inhibits pro-inflammatory macrophage activation [14]. Rerouting metabolic flux away from glycolysis cuts the metabolic support that M1 inflammatory activity requires and promotes polarization toward a reparative M2-like phenotype [28].

**Figure 1:**
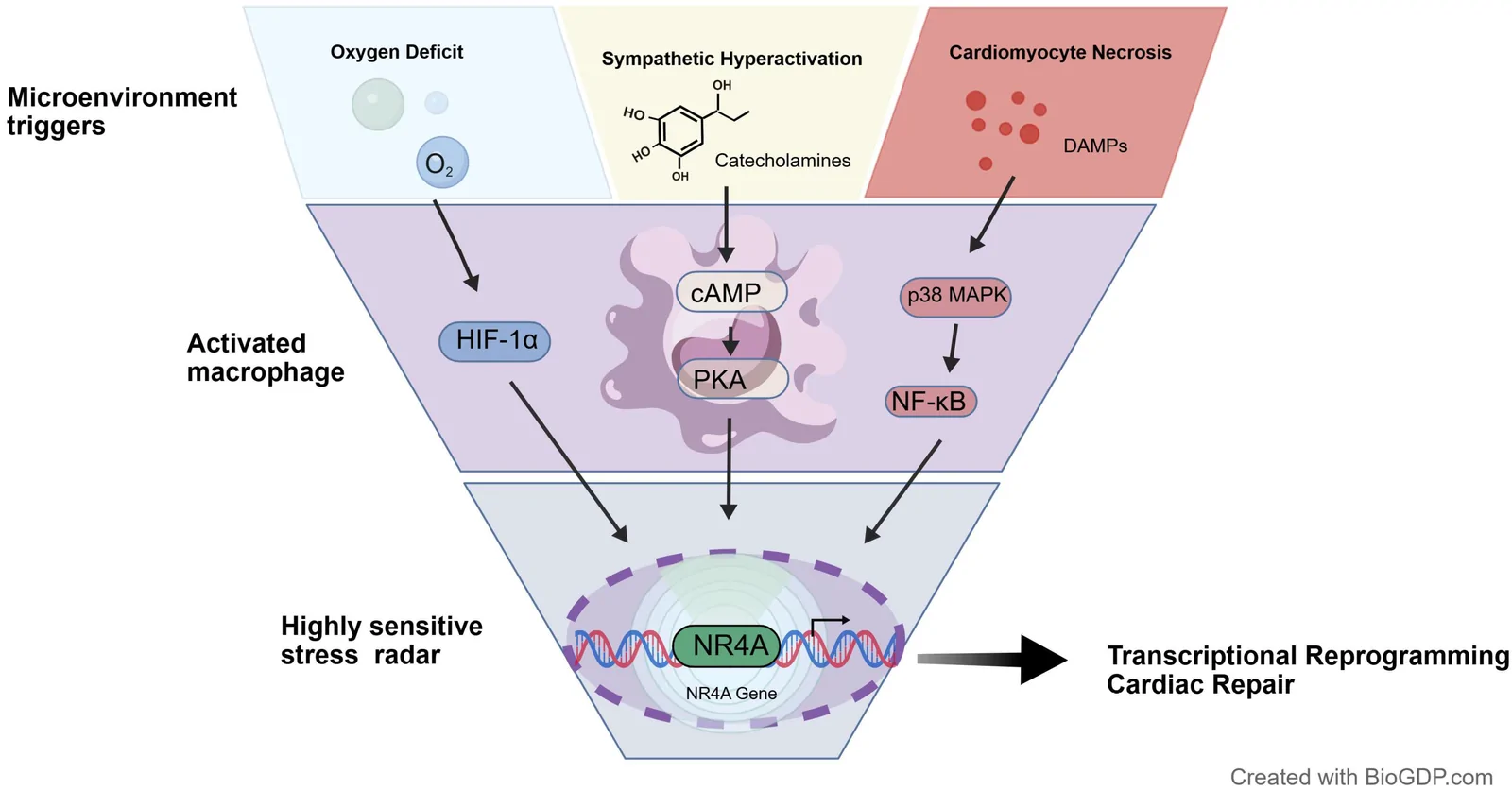
Schematic representation of NR4A signaling pathways proposed from preclinical and mechanistic studies. Microenvironmental cues associated with myocardial injury can activate HIF-1α, cAMP/PKA, and p38 MAPK–NF-κB pathways, thereby inducing NR4A expression and influencing macrophage transcriptional programs. Arrows indicate the direction of signaling pathways and regulatory relationships. Colors are used only for visual distinction and do not represent specific biological categories. Created with BioGDP.com. Abbreviations: HIF-1α, hypoxia-inducible factor 1 alpha; cAMP, cyclic adenosine monophosphate; PKA, protein kinase A; MAPK, mitogen-activated protein kinase; NF-κB, nuclear factor kappa B; DAMPs, damage-associated molecular patterns; NR4A, nuclear receptor subfamily 4 group A.

Transcriptionally, NR4A works through two routes: direct transactivation and indirect transrepression [15]. In direct transactivation, the receptors act as monomers or homodimers and bind specific DNA response elements—NR4A-binding elements (NBREs) or Nur response elements (NurREs)—located within the promoter regions of target genes [15]. This binding activates transcription of anti-inflammatory cytokines such as interleukin-10 (IL-10) and transforming growth factor-β (TGF-β), as well as genes encoding tissue remodeling factors, which together promote debris clearance and tissue repair [28]. In a murine model of myocardial infarction, cluster of differentiation 36 (CD36)-mediated phagocytosis of dying cardiomyocytes was associated with induction of Nr4a1 in recruited cardiac monocytes and with their subsequent transition toward a reparative phenotype. That work further identified Nr4a1 as a regulator of the efferocytic receptor Mer receptor tyrosine kinase (MerTK), supporting a CD36–Nr4a1–MerTK axis that links efferocytosis to macrophage-mediated cardiac repair [29]. NR4A receptors also modulate NF-κB-dependent inflammatory transcription, although the direction and underlying mechanisms appear to vary among NR4A family members and cellular contexts. In macrophage and myeloid-cell models, NR4A signaling has been implicated in the regulation of NF-κB-driven inflammatory responses [30,31]. Compensatory expression of NR4A1 (Nur77) and NR4A2 (Nurr1) has also been shown to regulate NF-κB-dependent inflammatory signaling in astrocytes [32]. NR4A1 (Nur77) can suppress inflammatory signaling by limiting NF-κB p65 binding to κB-responsive DNA elements [33]. A REST corepressor (CoREST)-dependent transrepression mechanism, by contrast, has been characterized most directly for NR4A2 (Nurr1) in activated microglia and astrocytes, where NR4A2 (Nurr1) associates with promoter-bound NF-κB p65 and recruits the CoREST corepressor complex to repress inflammatory gene transcription [34]. Metabolic reprogramming and epigenetic repression thus act together on the sterile inflammatory response mediated by monocytes/macrophages, tipping the post-ischemic environment toward inflammation resolution and structural regeneration [35]. The microenvironmental activation and downstream regulatory mechanisms of NR4A signaling are summarized in Figure 1.

## 4. The Role of Macrophage Subsets and Their Regulatory Drivers in Post-MI Remodeling

**Figure 2:**
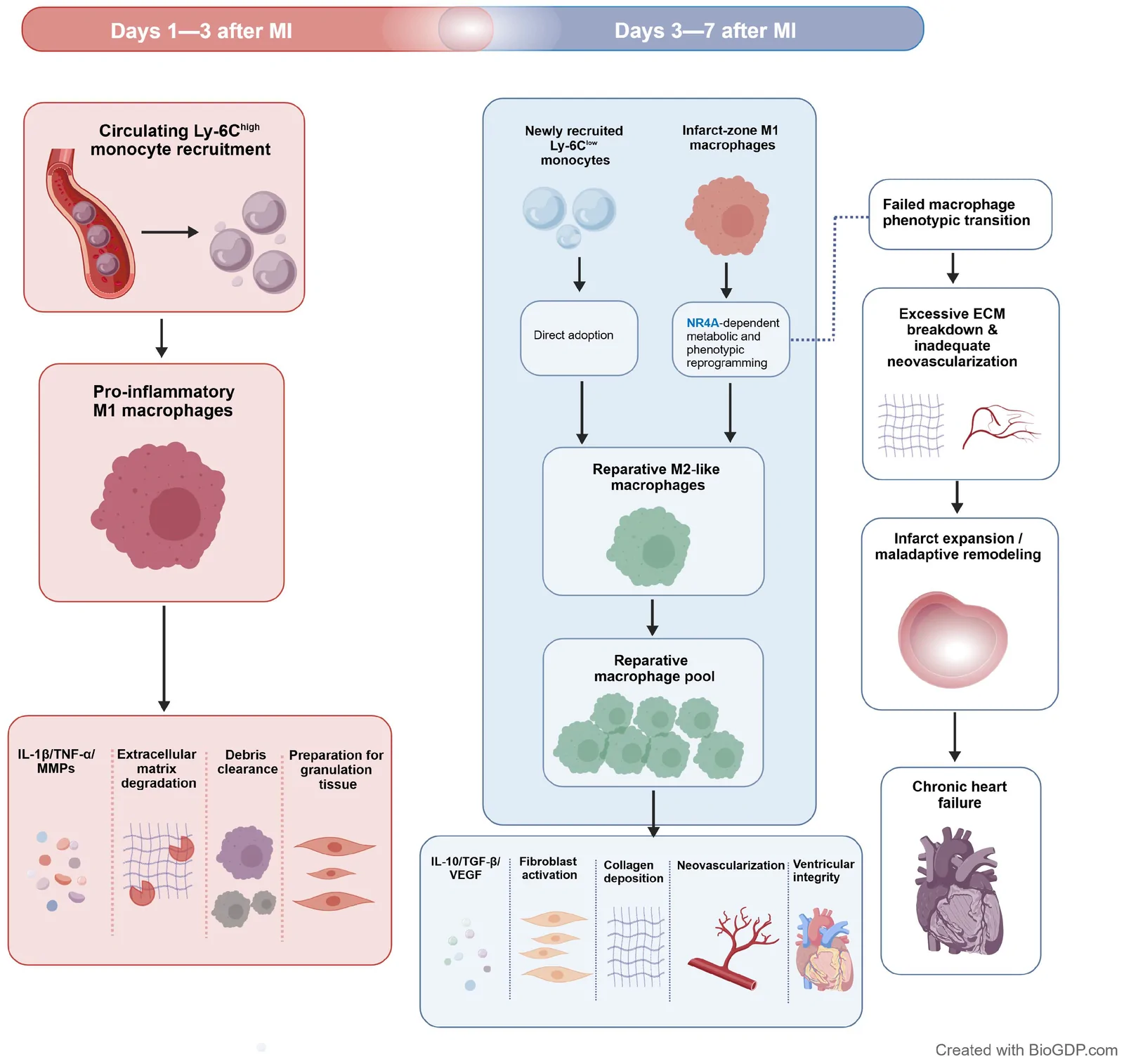
Temporal dynamics of macrophage polarization and consequences for post-infarct remodeling. In experimental murine models of myocardial infarction, Ly-6C^high^ monocyte-derived M1 macrophages initially clear necrotic tissue and degraded matrix, whereas subsequent reprogramming of resident M1 cells and recruitment of Ly-6C^low^ monocytes generate a reparative M2-like pool that supports collagen deposition, neovascularization, and ventricular stability; disruption of this transition results in persistent inflammation, adverse remodeling, and chronic heart failure. Arrows indicate the progression of macrophage responses, and the colors distinguish the inflammatory and reparative phases after MI. Created with BioGDP.com.

Tissue repair after MI depends on the spatiotemporal dynamics of diverse macrophage subsets [36,37]. Experimental murine models of myocardial infarction have revealed a characteristic spatiotemporal sequence of monocyte/macrophage responses during infarct healing. Within 1–3 days following coronary artery occlusion, extensive cardiomyocyte necrosis releases substantial amounts of DAMPs into the local microenvironment and triggers a strong sterile inflammatory response [38]. These DAMPs activate endothelial cells and resident immune cells to produce chemokine gradients, which in turn attract circulating Ly-6C^high^ monocytes into the damaged heart, where they differentiate rapidly into a pro-inflammatory M1 macrophage phenotype [22,39,40]. These newly differentiated M1 macrophages secrete inflammatory mediators, including interleukin-1 beta (IL-1β), tumor necrosis factor-alpha (TNF-α), and multiple matrix metalloproteinases (MMPs), which degrade the damaged extracellular matrix and clear cellular debris, thereby preparing the microenvironment for granulation tissue formation [41]. Between days 3 and 7 post-coronary artery occlusion, the local microenvironment transitions toward resolution of inflammation [3]. During this transitional phase, the M1 macrophages already present in the infarct zone undergo extensive in situ metabolic and phenotypic reprogramming, gradually transitioning toward a reparative M2-like phenotype. Concurrently, newly recruited Ly-6C^low^ monocytes from the circulation enter the damaged tissue and directly adopt an M2-like reparative phenotype [10,42]. Together, these two distinct cellular sources establish the reparative macrophage pool that drives subsequent tissue healing [3]. These reparative M2 cells suppress inflammatory signaling and release factors such as IL-10, TGF-β, and vascular endothelial growth factor (VEGF), which promote tissue recovery [9,10]. Therefore, they activate fibroblasts to promote collagen deposition and neovascularization, thus preserving the structural and mechanical integrity of the ventricle [43]. The temporal dynamics of macrophage subsets during post-infarction inflammation and repair are summarized in Figure 2.

Macrophage phenotypic switching is greatly affected by any disturbance of the process and consequently has an important effect on cardiac remodeling [44]. This balance is disturbed by dysfunctional polarization of macrophages, which causes excessive matrix degradation and poor neovascularization [12,41]. Once the regulatory mechanisms that control the heterogeneity of macrophages are disturbed, infarct expansion and pathological ventricular remodeling will occur, and it will become a critical factor in the development of irreversible chronic heart failure [45].

Since polarization, together with macrophages, plays a central role in cardiac repair, the determining factors of macrophage plasticity are currently an area of cardiovascular immunology research [36]. Of these, the NR4A family of orphan nuclear receptors has become a master regulator [19]. NR4A signaling is a molecular connection between the inflammatory and metabolic–epigenetic pathways that are active in the extracellular space, and it represents a critical point of decision to either promote or limit the progression of the immune microenvironment to repair and halt chronic injury [14]. This recognition leads to the subsequent study of the details of how NR4A allows macrophage-mediated cardioprotection.

It should be noted that the M1/M2 dichotomy, first proposed to describe macrophage activation states under defined in vitro stimulation conditions, represents a simplified operational framework rather than a complete description of macrophage biology in vivo. Recent single-cell and spatial transcriptomic studies of the infarcted human and murine heart place cardiac macrophages along a continuum of transcriptionally distinct states rather than in two discrete populations, with functional signatures that extend beyond the conventional M1/M2 classification [46,47]. We retain the M1/M2 terminology throughout this review for consistency with the original studies cited, while acknowledging that the framework is a simplification. How NR4A signaling shapes this finer-grained, in vivo heterogeneity of reparative macrophage subsets remains an important open question for future single-cell-resolution studies.

## 5. Molecular Mechanisms and Reparative Outcomes of NR4A-Regulated Macrophages

The mechanistic evidence discussed in this section is derived predominantly from preclinical studies, including cultured macrophage systems and experimental cardiovascular disease models, with direct validation in human myocardial infarction remaining limited. The NR4A family functions as an integrated regulatory network that governs macrophage phenotypic plasticity and cardiac repair after MI, with NR4A1 (Nur77) being the most extensively studied member [16]. NR4A1 (Nur77) acts as a key “molecular brake” that orchestrates a profound transition from pro-inflammatory M1 macrophages to pro-reparative M2 macrophages, thereby critically influencing cardiac healing trajectories [30,48]. Mechanistically, this phenotypic transition arises from metabolic and epigenetic reprogramming [49]. Mechanistic studies in experimental macrophage systems indicate that NR4A1 (Nur77) can shift cellular metabolism away from a strongly glycolytic inflammatory state toward oxidative metabolism [14,50]. Concurrently, NR4A1 (Nur77) can restrain NF-κB-dependent inflammatory signaling by limiting p65 binding to κB-responsive DNA elements [33]. A distinct CoREST-dependent transrepression mechanism has been demonstrated for NR4A2 (Nurr1) in activated microglia and astrocytes. In these cells, NR4A2 (Nurr1) associates with promoter-bound NF-κB p65 and recruits the CoREST corepressor complex, thereby suppressing transcription of pro-inflammatory genes [34]. Importantly, this mechanism has been demonstrated in activated microglia and astrocytes rather than in macrophages after myocardial infarction. Whether NR4A1 (Nur77) similarly recruits CoREST to suppress NF-κB-dependent transcription in post-MI macrophages therefore remains to be established.

The interaction between macrophages and the microenvironment of the necrotic tissue further promotes the NR4A1-mediated reparative differentiation process [51,52]. After the infarction, the large number of dead cardiomyocyte remnants would trigger the removal of apoptotic and necrotic material and thus help to dampen the inflammatory environment [53]. Apoptotic body clearance is mediated by the formation of phagosomes that serve as a functional signal to stimulate NR4A1 expression, launching a feed-forward reparative pathway [29,53]. This increase in NR4A1 prevents inflammatory signaling in the short term but promotes the release of long-term anti-inflammatory mediators to promote the resolution of sterile inflammation [54].

Functionally, preclinical studies indicate that NR4A1 (Nur77) can influence cardiac remodeling and macrophage functions relevant to tissue repair. In experimental cardiovascular models, NR4A1 (Nur77) has been implicated in limiting adverse cardiac remodeling [55]. Experimental systems have also demonstrated NR4A1-dependent regulation of fibrotic signaling and extracellular matrix homeostasis, while NR4A1 (Nur77) deficiency alters inflammatory responses, extracellular matrix-related programs, and phagocytic functions in bone marrow-derived macrophages [56,57]. A wider body of preclinical evidence, not restricted to studies examining NR4A1 (Nur77) directly, demonstrates that reparative macrophages can support cardiac repair through several complementary mechanisms. In experimental models of acute myocardial infarction, macrophages have been shown to contribute to cardioprotective responses and myocardial recovery [58]. In cell-based hypoxia/reoxygenation models, M2 macrophage-derived exosomes can protect cardiomyocytes from injury through the transfer of regulatory microRNAs [59]. Similarly, M2 macrophage-derived exosomal signaling has been implicated in attenuation of myocardial ischemia–reperfusion injury in preclinical experimental systems [60]. Efferocytosis-driven metabolic reprogramming, including enhanced fatty acid oxidation and electron transport, likewise promotes macrophage polarization toward tissue-reparative states [61]. These studies provide mechanistic support for the cardioprotective potential of reparative macrophages, but they should not be interpreted as direct evidence that all of these effects are specifically mediated by NR4A1 (Nur77).

The biological effects of NR4A1 (Nur77) are context-dependent and should not be regarded as uniformly anti-inflammatory or reparative. In atherosclerotic models, NR4A1 (Nur77) deficiency promotes a pro-inflammatory macrophage phenotype and accelerates lesion development, which supports a protective role for NR4A1 (Nur77) in the vascular inflammatory environment [19]. A murine model of acute myocardial infarction points to the same conclusion: Nr4a1 contributes to the balance between inflammatory and reparative monocyte/macrophage responses, and its deficiency impairs infarct healing and cardiac function [22]. The opposite is seen in an angiotensin II-induced mouse model of non-ischemic cardiac fibrosis, where Nr4a1 deletion reduced fibrosis and preserved diastolic function, accompanied by a shift away from pro-inflammatory macrophage and pro-fibrotic fibroblast phenotypes [62]. These apparently divergent observations indicate that the effects of NR4A1 (Nur77) depend on the tissue environment, cellular compartment, nature and duration of the stimulus, and stage of disease. The therapeutic consequences of NR4A1 (Nur77) modulation cannot, then, be assumed to be uniformly beneficial across different cardiovascular settings.

In addition to NR4A1 (Nur77), the other members of the NR4A family (NR4A2/Nurr1 and NR4A3/NOR-1) have been shown to regulate metabolic and epigenetic remodeling in macrophages, but little work has been done in the context of MI. NR4A2 (Nurr1) can regulate the phenotypes of macrophages via intersecting transcriptional networks with metabolic pathways, affecting lipid metabolism and inflammatory responses in atherosclerosis; notably, this includes evidence from human atherosclerotic lesion macrophages, in which NR4A1 (Nur77), NR4A2 (Nurr1), and NR4A3 (NOR-1) expression were associated with reduced lipid loading and inflammatory responses [18,63], underscoring its potential relevance to human cardiac repair signaling. NR4A3 (NOR-1) is associated with vascular smooth muscle cell proliferation and spatial regulation of immunocytes during vascular injury and may thus be involved in regulation of macrophage dynamics in the microenvironment of the infarct by playing a role in matrix remodeling and migration of immunocytes [20].

To summarize, while NR4A1 (Nur77) is the current focus of research, the NR4A family in general is crucial for the integration of extracellular inflammatory signals and intracellular metabolic–epigenetic programs, hence regulating macrophage plasticity and cardiac remodeling [15]. However, the specific roles of NR4A2 (Nurr1) and NR4A3 (NOR-1) in the regulation of the polarization of macrophages during myocardial infarction remain poorly understood and are important areas for future research. Representative experimental evidence discussed in this review, including the experimental model and biological context, NR4A family member, proposed mechanism, functional outcome, and strength of inference, is summarized in Table 1.

## 6. The Therapeutic Potential of NR4A in Broad Immune–Metabolic Diseases

Beyond the context of myocardial infarction, the NR4A family plays important roles in immune–metabolic regulation across a range of immune-related diseases and pathological microenvironments, highlighting its broader therapeutic potential [65].

In the tumor microenvironment, chronic exposure to antigens leads to the upregulation of NR4A in CD8+ T cells, which helps to gradually exhaust them [66]. Preclinical tumor studies have shown that disruption of NR4A signaling in chimeric antigen receptor T (CAR-T) cells can enhance antitumor effector function [66,67]. In murine atherosclerosis models, genetic loss of NR4A1 (Nur77) in hematopoietic or bone marrow-derived cells promotes a pro-inflammatory macrophage phenotype and exacerbates atherosclerotic lesion development [19,64]. These experimental findings have a human counterpart: NR4A family members (NR4A1, NR4A2, and NR4A3) are expressed in macrophages within human atherosclerotic plaques, and functional studies using human macrophages in vitro have demonstrated reduced lipid loading and inflammatory responses following NR4A overexpression [63]. In obesity-induced metabolic syndrome, NR4A has been proposed to regulate the polarization of adipose tissue macrophages (ATMs) and to prevent their transition to a pro-inflammatory, endocrine-disturbing profile. However, direct evidence that NR4A regulates ATM polarization in obesity is still lacking. It is well established that NR4A activation reduces macrophage-mediated metabolic inflammation, suggesting a potential role for NR4A in regulating immune homeostasis in adipose tissue [68,69]. Given that NR4A inhibits chronic metabolic inflammation at its source, it may be a promising pharmacological target for improving systemic insulin resistance in the future [69]. For instance, whereas NR4A1 (Nur77) suppresses scavenger receptor expression in the chronic, lipid-laden environment of atherosclerotic plaques, it is induced by efferocytosis-driven signals during the acute post-MI clearance phase, illustrating how the same receptor family can drive divergent transcriptional programs depending on the surrounding stimulus.

Direct human evidence specifically linking NR4A signaling in macrophages to myocardial infarction remains limited. Bonta et al. reported that NR4A1 (Nur77), NR4A2 (Nurr1), and NR4A3 (NOR-1) are expressed in human atherosclerotic lesion macrophages and are associated with reduced lipid loading and inflammatory responses, providing human tissue-level evidence for NR4A-related macrophage biology [63]. High-resolution single-cell and spatial transcriptomic atlases of human infarcted myocardium have recently become available [46,47]. However, whether NR4A-associated transcriptional programs are present in macrophage subsets after human myocardial infarction has not yet been directly established. Analysis of these existing datasets may help evaluate the translational relevance of the mechanisms reviewed here.

## 7. Future Perspectives and Hurdles of NR4A as a Target

Current evidence supporting therapeutic modulation of NR4A in cardiovascular disease remains predominantly preclinical. Since NR4A receptors do not have a typical ligand-binding cavity, they have been labeled as “undruggable” targets [70,71]. That has been shaken in recent years by advances in structural characterization and extensive screening of compounds; however, tangible routes towards clinical application have been found [70]. Currently, the NR4A family is targeted by two types of intervention strategy: small molecule modulators and precision nanomedicine delivery systems [72]. Pharmacologically, both natural and synthetic allosteric agonists like cytosporone B (Csn-B) and the pyrrolidinedithiocarbamate (PDC)-derivative novel peptide analog (PDNPA) have shown potent effects in reducing interstitial fibrosis and preventing the pro-inflammatory cascades mediated by macrophages in post-MI ventricular remodeling [73]. However, the systemic use of small-molecule NR4A agonists carries documented safety liabilities: Csn-B has been shown to elevate fasting blood glucose in mice by inducing gluconeogenic gene expression [74]. This metabolic effect is of particular concern, given the frequent co-existence of impaired glucose tolerance in MI patients. More broadly, the systemic use of small molecules is also hampered by off-target effects and pharmacokinetic issues that can affect both therapeutic efficacy and safety.

Preclinical studies have demonstrated that macrophage-targeted liposomal or nucleic-acid-based approaches can modulate macrophage phenotype and improve infarct healing in experimental MI models [75,76]. These studies provide proof of concept for macrophage-directed therapeutic delivery; however, direct NR4A1-specific mRNA or DNA delivery to cardiac macrophages remains a prospective strategy rather than an established therapeutic approach. However, nanoparticle- and exosome-based delivery platforms carry class-recognized safety liabilities, including immunogenicity, complement activation, and off-target accumulation in non-target organs, which warrant careful evaluation before clinical translation [77]. Direct delivery of NR4A1-encoding mRNA or plasmid DNA to cardiac macrophages holds promise for precisely inducing reparative M2-phenotype macrophages via in situ reprogramming, an emerging approach that can confer therapeutic benefits while reducing systemic immunosuppression [78].

However, NR4A-based therapies still face three major challenges before their widespread clinical application [70]. First, tissue pleiotropy raises oncological safety concerns: systemic activation of NR4A receptors may inadvertently promote tumor cell survival or contribute to cluster of differentiation 8-positive (CD8+) T cell exhaustion, potentially undermining antitumor immunity [79]. Second, NR4A1 (Nur77), NR4A2 (Nurr1), and NR4A3 (NOR-1) share a high degree of sequence similarity in their ligand-binding domains (LBDs), thereby increasing drug-design complexity and making subtype-selective targeting difficult; this is of particular concern because non-selective pan-NR4A agonists may inadvertently activate NR4A2 (Nurr1), which plays an essential role in the survival and maintenance of dopaminergic neurons, raising a theoretical risk of unintended central nervous system effects with systemic, chronic NR4A-targeted therapy [80]. The third is timing. Because the initial M1-dominant phase of the post-MI immune response is required for efficient clearance of necrotic debris, premature or excessive activation of NR4A signaling could theoretically blunt this necessary early phagocytic response, potentially delaying wound healing or increasing the risk of ventricular rupture; precise temporal control is therefore essential to any therapeutic strategy targeting NR4A [9].

NR4A-mediated immunomodulation remains a promising therapeutic strategy for cardiac repair, but several biological and pharmacological challenges stand in the way. Understanding the structure of NR4A, designing new selective ligands for it, and advancing ligand delivery technologies will all be needed before the translational potential of the NR4A family can be fully realized.

## 8. Conclusions

Collectively, preclinical evidence supports NR4A signaling as a promising regulatory axis linking macrophage inflammatory resolution, metabolic reprogramming, and tissue repair after myocardial injury. However, much of the mechanistic and therapeutic evidence currently derives from experimental cell and animal models, and direct validation in human myocardial infarction remains limited. Further studies using human cardiac tissues, clinically relevant models, and carefully designed translational approaches will therefore be required before the therapeutic potential of NR4A targeting can be established.

## Figures and Tables

**Table 1:** Representative experimental evidence for NR4A-mediated regulation of macrophage phenotype in cardiovascular disease and cardiac repair.

Model System/ Experimental Context	NR4A Member	Proposed Mechanism/Key Mechanistic Finding	Functional/Reparative Outcome	Strength of Inference ^1^	Ref.
*Nr4a1−/−* mice in an atherosclerosis model; macrophage assays	NR4A1 (Nur77)	Nr4a1 deficiency promotes a pro-inflammatory macrophage phenotype and enhances inflammatory signaling	Increased atherosclerotic lesion development and vascular inflammation	Strong	[19]
*Ldlr−/−* mice reconstituted with *Nur77−/−* bone marrow; murine BMDMs	NR4A1 (Nur77)	Loss of myeloid NR4A1 (Nur77) enhances inflammatory macrophage activation and inflammatory monocyte/macrophage recruitment	Increased atherosclerotic lesion size, inflammatory-cell accumulation, and necrotic-core formation	Strong	[64]
*Nr4a1−/−* mice, experimental myocardial infarction	NR4A1 (Nur77)	Nr4a1 contributes to the balance between inflammatory and reparative monocyte/macrophage responses after MI	Disrupted inflammation–repair balance and impaired infarct healing in the absence of Nr4a1	Strong	[22]
Murine myocardial infarction model with impaired myeloid CD36 signaling; macrophage assays	NR4A1 (Nur77)	CD36-mediated phagocytosis promotes Nr4a1-associated reparative programming, while Nr4a1 contributes to regulation of the efferocytic receptor MerTK	Impaired efferocytosis and reparative monocyte/macrophage transition when the CD36–Nr4a1 pathway is disrupted	Moderate	[29]
Murine BMDMs with complementary in vivo atherosclerosis experiments	NR4A1 (Nur77)	NR4A1 (Nur77) restrains pro-inflammatory metabolic reprogramming by regulating mitochondrial metabolism and succinate/SDH-associated inflammatory signaling	Reduced pro-inflammatory cytokine production under intact NR4A1 (Nur77) signaling; NR4A1 (Nur77) deficiency aggravates inflammatory responses	Moderate	[14]
WT and *Nr4a1−/−* mice, angiotensin II-induced nonischemic cardiac fibrosis; macrophage and fibroblast assays	NR4A1 (Nur77)	In this stimulus context, Nr4a1 promotes pro-inflammatory macrophage and pro-fibrotic fibroblast responses and contributes to macrophage–fibroblast crosstalk	NR4A1 (Nur77) deletion reduces cardiac fibrosis and preserves diastolic function	Strong	[62]
Human atherosclerotic plaques and human macrophages studied in vitro	NR4A1/NR4A2/NR4A3	NR4A receptors are expressed in human plaque macrophages and functionally limit macrophage lipid loading and inflammatory responses	Reduced lipid accumulation and inflammatory mediator production in macrophage functional assays	Moderate	[63]

Abbreviations: MI, myocardial infarction; BMDM, bone marrow-derived macrophage; SDH, succinate dehydrogenase; WT, wild type. ^1^ Strength of inference refers to the extent to which the experimental design supports a causal relationship between NR4A signaling and the reported phenotype, rather than overall study quality. “Strong” indicates direct genetic manipulation of the relevant NR4A pathway in an in vivo disease model with concordant phenotypic evidence; “moderate” indicates mechanistic perturbation in vitro or interventional evidence with limitations such as incomplete physiological context or indirect manipulation of the NR4A pathway. Gene symbols and genotypes (e.g., *Nr4a1−/−* and *Ldlr−/−*) are italicized, whereas protein names are presented in roman type.

## Data Availability

No new data were created or analyzed in this study. Data sharing is not applicable to this article.

## Abbreviations

The following abbreviations are used in this manuscript:

NR4A	Nuclear receptor subfamily 4 group A
NF-κB	Nuclear factor kappa B pathway
MI	Myocardial infarction
IL-4	Interleukin-4
HREs	Hypoxia response elements
DAMPs	Damage-associated molecular patterns
IFN-γ	Interferon gamma
LPS	Lipopolysaccharide
IL-1β	Interleukin-1 beta
IL-10	Interleukin-10
I/R	Ischemia–reperfusion
MAPK	Mitogen-activated protein kinase
cAMP	Cyclic adenosine monophosphate
MMPs	Matrix metalloproteinases
PKA	Protein kinase A
NR4A1	Nuclear receptor subfamily 4 group A member 1
NR4A2	Nuclear receptor subfamily 4 group A member 2
NR4A3	Nuclear receptor subfamily 4 group A member 3
OXPHOS	Oxidative phosphorylation
SDH	Succinate dehydrogenase
Csn-B	Cytosporone B
TGF-β	Transforming growth factor beta
TLRs	Toll-like receptors
TNF-α	Tumor necrosis factor alpha
VEGF	Vascular endothelial growth factor
NBREs	NR4A-binding elements
NurREs	Nur response elements
MerTK	Mer receptor tyrosine kinase
CoREST	REST corepressor
CAR-T	Chimeric antigen receptor T
PDC	Pyrrolidinedithiocarbamate
PDNPA	Pyrrolidinedithiocarbamate-derivative novel peptide analog
CD8+ T	Cluster of differentiation 8-positive (CD8+) T
LBDs	Ligand-binding domains
